# Integrative network analysis of differentially methylated regions to study the impact of gestational weight gain on maternal metabolism and fetal-neonatal growth

**DOI:** 10.1590/1678-4685-GMB-2023-0203

**Published:** 2024-03-25

**Authors:** Perla Pizzi Argentato, João Victor da Silva Guerra, Liania Alves Luzia, Ester Silveira Ramos, Mariana Maschietto, Patrícia Helen de Carvalho Rondó

**Affiliations:** 1 Universidade de São Paulo, Faculdade de Saúde Pública, Departamento de Nutrição, São Paulo, SP, Brazil.; 2 Centro Nacional de Pesquisa em Energia e Materiais (CNPEM), Laboratório Nacional de Biociências (LNBio). Campinas, SP, Brazil.; 3 Universidade Estadual de Campinas, Faculdade de Ciências Farmacêuticas, Programa de Pós-Graduação em Ciências Farmacêuticas, Campinas, SP, Brazil.; 4 Universidade de São Paulo, Faculdade de Medicina de Ribeirão Preto, Departamento de Genética, Ribeirão Preto, SP, Brazil.; 5 Universidade Estadual de Campinas, Instituto de Biologia, Departamento de Biologia Estrutural e Funcional, Campinas, SP, Brazil.; 6 Centro Infantil Boldrini, Campinas, SP, Brazil.

**Keywords:** Gestational weight gain, DNA methylation, functional annotation, enrichment analysis, offspring growth

## Abstract

Integrative network analysis (INA) is important for identifying gene modules or epigenetically regulated molecular pathways in diseases. This study evaluated the effect of excessive gestational weight gain (EGWG) on INA of differentially methylated regions, maternal metabolism and offspring growth. Brazilian women from “The Araraquara Cohort Study” with adequate pre-pregnancy body mass index were divided into EGWG (n=30) versus adequate gestational weight gain (AGWG, n=45) groups. The methylome analysis was performed on maternal blood using the Illumina MethylationEPIC BeadChip. Fetal-neonatal growth was assessed by ultrasound and anthropometry, respectively. Maternal lipid and glycemic profiles were investigated. Maternal triglycerides-TG (p=0.030) and total cholesterol (p=0.014); fetus occipito-frontal diameter (p=0.005); neonate head circumference-HC (p=0.016) and thoracic perimeter (p=0.020) were greater in the EGWG compared to the AGWG group. Multiple linear regression analysis showed that maternal DNA methylation was associated with maternal TG and fasting insulin, fetal abdominal circumference, and fetal and neonate HC. The DMRs studied were enriched in 142 biological processes, 21 molecular functions,and 17 cellular components with terms directed for the fatty acids metabolism. Three DMGMs were identified:*COL3A1, ITGA4* and *KLRK1*. INA targeted chronic diseases and maternal metabolism contributing to an epigenetic understanding of the involvement of GWG in maternal metabolism and fetal-neonatal growth.

## Introduction

Pregnancy is associated with anatomical and physiological adjustments that lead to changes in the composition of blood cell and humoral elements. These changes begin at the time of implantation and persist throughout the gestational period. The uterus undergoes intense vascularization due to the need for greater blood perfusion. As pregnancy progresses, the placenta grows and uteroplacental blood flow increases, requiring a larger number of vessels (Kazma et al., 2020).

There are specific biochemical parameters for this period of life, which are suitable for the monitoring, diagnosis and prevention of diseases that can affect pregnant women and their fetus (Teasdale and Morton, 2018). In addition to these parameters, the assessment of the nutritional status and weight gain of pregnant women is important for their health and for fetal development, ensuring more favorable outcomes in prenatal care. The Institute of Medicine (IOM) recommends gestational weight gain based on pre-pregnancy body mass index (BMI) (Rasmussen and Yaktine, 2009).

Overweight is a global problem in women of reproductive age (Branum et al., 2016; Goldstein et al., 2018). Excess weight is associated with changes in inflammatory parameters, blood glucose, triglycerides, and lipids (Gulecoglu Onem et al., 2021; Rugină et al., 2021). Moreover, excessive weight gain during pregnancy can lead to preeclampsia and gestational diabetes, among other diseases (Girirajan et al., 2011), and can negatively affect fetal development and offspring health at different stages of life (Windham et al., 2019).

The development of chronic diseases in the offspring at different stages of life is explained by fetal metabolic programming, which describes the epigenetic mechanisms that modulate gene expression (Barker, 1998), such as DNA methylation (DNAm) (Godfrey et al., 2011). During pregnancy, epigenetic patterns of DNAm are associated with diseases such as obesity (Hjort et al., 2018), maternal diet (Thakali et al., 2020), smoking, alcoholism, and drug use (Subit and Mohammed, 2015). A growing number of studies using the Illumina Infinium Human MethylationEPIC BeadChip have generated data on pregnancy and fetal programming (Sharp et al., 2015; Hjort *et al*., 2018; Mas-Parés et al., 2023). A previous study from our research group showed that the maternal methylome of pregnant women with excessive gestational weight gain (EGWG) was altered at 46 differentially methylated positions and in 11 differentially methylated regions (DMRs) (Argentato et al., 2023). However, the literature on gestational weight gain using statistical tools designed for integrative network analysis is scarce. 

Integrated tools such as functional annotation and enrichment analysis are important for identifying gene modules or epigenetically regulated molecular pathways that play considerable roles in cell differentiation and diseases (Jiao et al., 2014). Therefore, the combination of the effect of EGWG on DNAm and functional enrichment analysis in women with adequate pre-pregnancy BMI, permits to explain how epigenetic changes may be implicated in maternal metabolism and fetal-neonatal growth.

This work aims to assess the effect of EGWG on: 1) the functional annotation and enrichment of differentially methylated regions (DMRs) and of differentially methylated gene modules (DMGMs) in women who had an adequate pre-pregnancy BMI 2) maternal metabolism and 3) offspring growth. 

## Material and Methods

### Subjects

This is a prospective cohort study involving 75 pregnant women from the “Araraquara Cohort Study”, Araraquara city, São Paulo, Brazil. Pregnant women with a normal pre-pregnancy BMI (≥18.5 and <24.9 kg/m^2^) were divided into two groups according to gestational weight gain recommended by the IOM (Rasmussen and Yaktine, 2009): EGWG (weight gain >16 kg; n=30) and adequate gestational weight gain (AGWG; weight gain > 11.5 kg and <16.0 kg; n=45). This study was conducted according to the guidelines of the Declaration of Helsinki and all procedures involving human subjects. All women and/or their legal guardian(s) signed the free informed consent form and the Research Ethics Committee of the Faculty of Public Health, University of São Paulo, approved in 12/05/2017 the study (protocol number 2.570.576), as described by Argentato et al. (2023).

We excluded pregnant women with more than 15 weeks of gestation, under 18 and over 35 years of age, with a pre-pregnancy BMI less than 18.5 kg/m^2^ (malnourished) or greater than 24.9 kg/m^2^ (overweight or obese), and diagnosed with chronic diseases, infectious diseases, severe mental illness, multiple pregnancy, history of miscarriage, smoking and use of alcohol or other drugs at the beginning of the study or during follow-up. Women who lost or gained little weight during pregnancy according to IOM (Rasmussen and Yaktine, 2009), who had a stillborn child or congenital diseases, or who did not attend one of the appointments during follow-up of the study were also excluded.

The women were evaluated at three different time points during pregnancy and at delivery: gestational age ≤ 15 weeks (T1); 20-26 weeks (T2), 30-36 weeks (T3), and delivery (T4). Gestational weight was assessed in the prenatal services at the three time points. The pre-pregnancy weight used was that measured before the 13th week of gestation. Weights at the three different time points during pregnancy and at delivery were measured with the Inbody 370 bioimpedance equipment (Biospace^®^, Seoul, Korea) by trained researchers using standardized procedures.

### Biological material

Maternal blood collection and biochemical analyses at T1, T2 and T3 were performed by a biomedical specialist in a clinical analysis laboratory in Araraquara city. The following parameters were analyzed: fasting glucose - FG (mg/dL), fasting insulin - FI (µIU/mL), total cholesterol - TC (mg/dL), triglycerides - TG (mg/dL), and high-sensitivity C-reactive protein-hs-CRP (mg/dL). A 2-mL aliquot of peripheral maternal blood was also collected at the end of pregnancy into a VACUETTE^®^ EDTA tube, homogenized manually, and refrigerated for DNA extraction and methylation analysis.

### Fetal growth

Fetal growth was evaluated at T2 and T3 by a trained sonographer with a Siemens ACUSON X300TM ultrasound system, premium edition (Siemens^®^, Mountain View, CA, USA), using abdominal curvilinear transducers (C5-2, C6-3, V7-3). The following fetal biometric measurements were assessed: biparietal diameter - BPD (cm), occipito-frontal diameter - OFD (cm), head circumference - HC (cm), abdominal circumference - AC (cm), femur length - FL (cm), humeral length - HL (cm), and length (cm).

### Neonatal anthropometry

After childbirth (T4), the neonates were weighed on a Soehnle Multina Plus electronic baby scale (Soehnle^®^, Germany). Length (cm) was measured with a Seca^®^ 416 infantometer (Seca^®^, Hamburg, Germany). In addition, HC (cm), thoracic perimeter - TP (cm), and AC (cm) were measured with a Seca^®^ 201 flexible tape (Seca^®^, Hamburg, Germany). To ensure accuracy and reproducibility of the measurements, the researchers attended a dedicated training course.

### Methylation analysis

A convenience sample of 16 DNA samples (EGWG, n=8 and AGWG, n=8), extracted from maternal blood at the end of gestation (T3) and matched for parity and neonete sex, was high-quality bisulfite-converted (EZ DNA Methylation Kit, Zymo Research Corp, Irvine, CA, USA) and submitted to preprocessing and analysis of methylation data with the Infinium MethylationEPIC BeadChip (850K) following the Illumina Infinium HD protocol at Diagenode (https://www.diagenode.com). The data were normalized using the beta-mixture quantile normalization (BMIQ) method (Teschendorff et al., 2013), corrected to batch effects variation (Johnson et al., 2007) and cellular composition (Houseman et al. 2012). Quality control assessment (Morris et al., 2014) was used to remove failed probes, probes with <3 beads in at least 5% of the samples, non-CG probes, multi-hit probes, and probes located in XYS (n=156,482), remaining 709,466 probes.

Singular value decomposition (SVD) analysis was applied to beta-values to correlate biological covariates with the principal components, as described by Argentato et al. (2023), which led to the identification of 11 DMRs between EGWG and AGWG (DMR1 = chr6:29648161-29648756, DMR2 = chr6:31148332-31148666, DMR3 = chr7:27183133-27183816, DMR4 = chr10:530635-531584, DMR5 = chr22:51016386-51016950, DMR6 = chr16:3062296-3062975, DMR7 = chr5:110062539-110062837, DMR8 = chr17:41278135-41278906, DMR9 = chr2:27301195-27301943, DMR10 = chr5:170288671-170288788, DMR11 = chr12:52281482-52281997) were identified. The mean beta value of the DMR using the beta-value of the CpG sites that compose the DMR was calculated.

The functional annotation of the DMRs was performed using GREAT (McLean et al., 2010). We defined annotations with a p-value <0.05 as enriched. The FEM package (Jiao et al., 2014) was applied to M-values, considering 100 seeds, 1,000 Monte Carlo runs and gamma of 0.5 for spin-glass algorithm, to identify differentially methylated gene modules (DMGMs). The enrichment analysis of DMGMs was done with the WebGestalt (Liao et al., 2019), considering Gene Ontology (Ashburner et al., 2000) and disease terms from the PharmGKB (Whirl-Carrillo et al., 2021). Genes associated with the individual disease were inferred using GLAD4U (Jourquin et al., 2012). Annotations with a p-value <0.05 were considered enriched.

### Data analysis

Descriptive statistics were used for data analysis. The Shapiro-Wilk test was applied to verify data normality. The *t*-test for independent samples was used for comparison between the EGWG and AGWG groups. Repeated measures ANOVA/mixed model with Bonferroni’s post-hoc was used, in which the follow-up measures were the repetition factor over time and the groups were the independent factor. Univariate and multiple linear regression models were used to explore the associations between average maternal DNAm level of each DMR and maternal biochemical parameters, fetal biometric measurements, and neonate anthropometry. The outcome measures were maternal FG, FI, TC, TG, and hs-CRP; fetal BPD, OFD, FL, HL, and length; fetal and neonate HC and AC, and neonate weight, length, and TP. The confounding variables included maternal age, pre-pregnancy BMI, gestational weight gain, gestational age, and neonate sex. Statistical significance was established at p<0.05. All analyses were performed using the SPSS software, version 18.0 (SPSS, Chicago, IL, USA).

## Results

### Biochemical parameters of the pregnant women, fetal growth and anthropometry of the neonate

**Figure 1 -:**
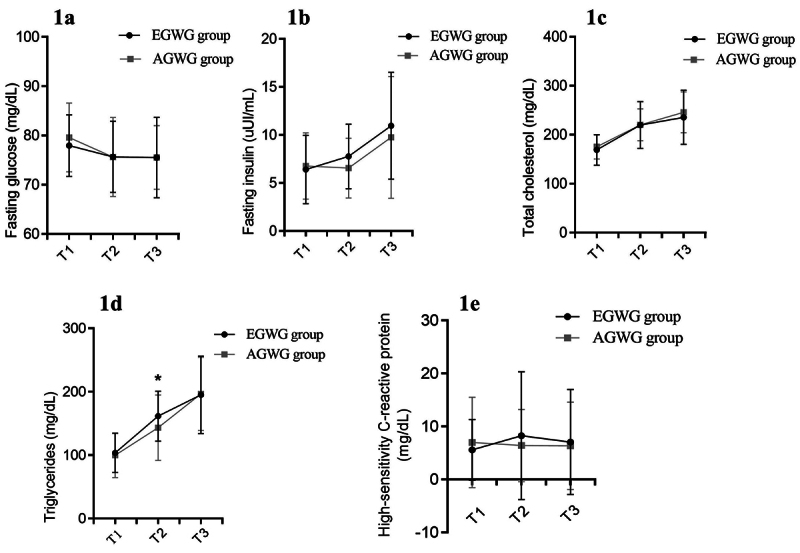
Evolution of the maternal biochemical parameters. Repeated measures ANOVA/mixed model with Bonferroni’s post hoc test. T1 = ≤ 15 gestational weeks, T2 = 20-26 weeks, T3 = 30-36 weeks, *p<0.05.

Regarding biochemical parameters, TG (p=0.030) and TC (p=0.014) were higher in the EGWG group compared to AGWG at T2, while no significant differences were observed for the other parameters. However, when these values were analyzed repeatedly, an effect of time on FG was found [F(2;146) = 37.12; p<0.001], with T1>T2 (p=0.035), T1>T3 (p<0.001). Time also had an effect on TC [F(2;146) = 176.33; p<0.001], with T3>T2>T1 (p<0.001). No group or time*group interaction effect on these parameters was found. There was an effect of time [F(2;146) = 210.53; p<0.001] on TG, with T3>T2>T1 (p<0.001), and an effect of time*group interaction [F(2;146) = 2.77; p=0.05], with T3>T2>T1 (p<0.001), in AGWG and EGWG. Comparison of the groups at each time point showed no differences at T1 or T3 (p=0.48 and p=0.86), while the biochemical parameters were higher at T2 in the EGWG group compared to AGWG (p=0.05). The evolution of the biochemical parameters is shown in Figure 1. Table 1 shows the maternal biochemical parameters in the AGWG and EGWG groups at T1, T2 and T3, fetal biometric measurements at T2 and T3, and neonate anthropometry at T4.

**Table 1 -:** Biochemical parameters of the pregnant women, biometric measurements of the fetus, and anthropometry of the neonates.

Variables	Excessive gestational weight gain (n= 30)	Adequate gestational weight gain (n= 45)	p
Pregnant women			
*Biochemical parameters*			
T1 Fasting blood sugar (mg/dL)	77.93±6.26	79.60±6.97	0.410
T2 Fasting blood sugar (mg/dL)	75.67±7.21	75.62±8.02	0.394
T3 Fasting blood sugar (mg/dL)	75.52±8.17	75.53±6.46	0.149
T1 Fasting insulin (uUI/mL)	6.40±3.56	6.98±4.27	0.718
T2 Fasting insulin (uUI/mL)	7.77±3.36	6.76±3.75	0.118
T3 Fasting insulin (uUI/mL)	10.97±5.56	9.56±6.01	0.199
T1 Total cholesterol (mg/dL)	169.10±31.21	175.38±24.87	0.106
T2 Total cholesterol (mg/dL)	220.27±32.45	219.87±47.77	0.014
T3 Total cholesterol (mg/dL)	235.66±55.15	245.82±41.74	0.408
T1 Triglycerides (mg/dL)	103.63±31.00	99.76±35.05	0.452
T2 Triglycerides (mg/dL)	161.50±39.30	143.29±51.58	0.030
T3 Triglycerides (mg/dL)	195.14±61.01	196.67±57.68	0.965
T1 Ultra-sensitive C-reactive protein (mg/dL)	6.98±8.54	5.60±5.74	0.129
T2 Ultra-sensitive C-reactive protein (mg/dL)	6.41±6.81	8.26±12.06	0.710
T3 Ultra-sensitive C-reactive protein (mg/dL)	6.35±8.22	7.06±9.91	0.658
Fetus			
*Biometric measurements*			
T2 Biparietal diameter (cm)	5.92±0.67	5.82±0.68	0.846
T3 Biparietal diameter (cm)	8.48±0.48	8.47±0.52	0.653
T2 Occipito-frontal diameter (cm)	7.48±0.76	7.44±0.81	0.423
T3 Occipito-frontal diameter (cm)	10.50±0.51	10.71±0.85	0.005
T2 Head circumference (cm)	21.20±2.16	21.23±2.91	0.978
T3 Head circumference (cm)	29.97±1.39	29.93±3.79	0.099
T2 Abdominal circumference (cm)	18.44±2.20	18.46±2.41	0.508
T3 Abdominal circumference (cm)	28.34±1.78	28.79±2.25	0.176
T2 Femur length (cm)	4.10±0.63	4.03±0.58	0.897
T3 Femur length (cm)	6.28±0.40	6.27±0.44	0.770
T2 Humeral length (cm)	3.87±0.53	3.77±0.45	0.338
T3 Humeral length (cm)	5.52±0.39	5.56±0.42	0.283
T2 Length (cm)	28.71±4.35	28.14±4.19	0.938
T3 Length (cm)	43.90±2.81	43.77±3.26	0.928
Neonate			
*Anthropometry*			
T4 Weight (g)	3502.50±292.45	3213.00±418.33	0.109
T4 Length (cm)	50.03±1.78	48.80±2.33	0.182
T4 Head circumference (cm)	34.57±1.30	33.68±1.44	0.016
T4 Thoracic perimeter (cm)	33.98±1.55	33.12±1.64	0.020
T4 Abdominal circumference (cm)	32.58±1.94	30.89±2.38	0.256

Mean±SD. T test for independent samples. BMI: Body Mass Index. T1 = ≤ 15 gestational weeks. T2 = 20-26 weeks. T3 = 30-36 weeks and T4 = delivery.

Regarding fetal growth, a difference in the biometric measurements between the two groups was observed for OFD at T3, which was lower in EGWG compared to AGWG (p=0.005). There was no significant difference in the other biometric measurements investigated. However, when the fetal growth parameters were analyzed repeatedly, there was an effect of time on OFD [F(1;73) = 748.84; p<0.001], BPD [F(1;73) = 957.91; p<0.001], FL [F(1;73) = 998.19; p<0.001], and HL [F(1;73) = 657.81; p<0.001], with T3>T2 (p<0.001 for all parameters). For the evolution of growth see Figure S1. There was no group or time*group interaction effect on these parameters.

**Figure 2 -:**
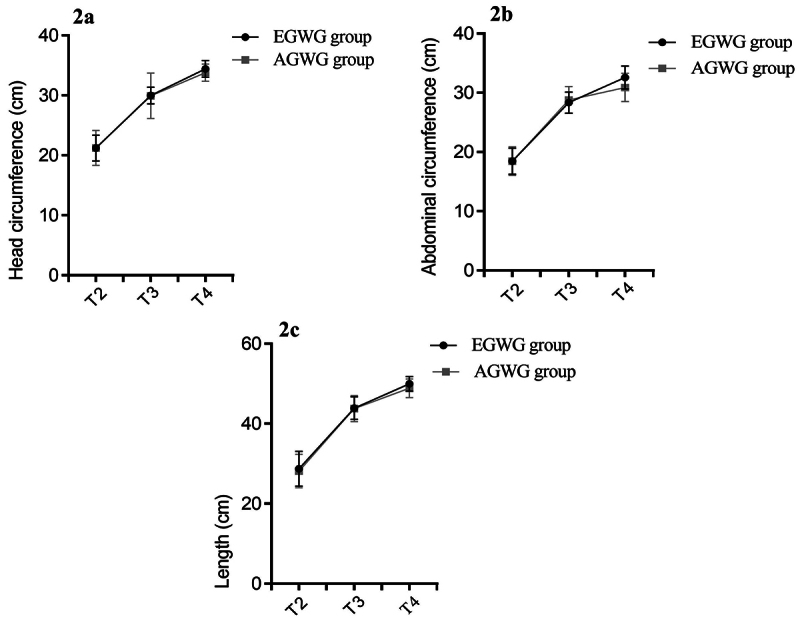
Evolution of the offspring growth parameters. Repeated measures ANOVA/mixed model with Bonferroni’s post hoc test. T1 = ≤ 15 gestational weeks. T2= 20-26 weeks. T3= 30-36 weeks and T4= delivery.

With respect to neonate anthropometry, HC (p=0.016) and TP (p=0.020) were higher in the EGWG group compared to AGWG. However, no differences were found for the other parameters. When HC, AC and length were compared repeatedly, there was an effect of time on HC [F(2;146) = 1279.33; p < 0.001], with T4>T3>T2 (p<0.001), on AC [F(2;146) = 872.45; p<0.001], with T4>T3>T2 (p<0.001), and on length [F(2;146) = 1144.72; p<0.001], with T4>T3>T2 (p<0.001). The evolution of the growth parameters is presented in Figure 2. There was no effect of group or time*group interaction on these parameters.

### Multiple linear regression models with the maternal DNA methylation

The biological covariates shown in Table 1 were also correlated with the principal components of the methylation data. After singular value decomposition (SVD) deconvolution, which corrected the batch effects variation, the first principal component of variation (PC-1) in the dataset was associated with three biological factors of interest, hs-CRP in T3, length in T3, and FL in T3; p<0.05). Therefore, PC-1 captured about 56% of the variance in the dataset. Likewise, the significant biological covariates identified by SVD analysis showed slightly different patterns in each group, indicating that hs-CRP in T3, length In T3, and FL in T3 may be related to the epigenetic signature.

We explored the associations between the mean maternal DNAm level of each DMR and maternal biochemical parameters, fetal growth parameters and neonate anthropometry. There were associations between DMR1 and HC (p=0.023) at T4; DMR2 and FI (p=0.001) at T2; DMR2 and TG (p=0.034) at T1; DMR2 and TG (p=0.029) at T2, DMR3 and AC (p=0.006) at T3; DMR5 and AC (p=0.009) at T3; DMR5 and HC (p=0.001) at T3; DMR6 and FI (p=0.008) at T2; DMR9 and TG (p=0.001) at T3, and DMR9 and AC (p=0.046) at T3. The significant model associations between the mean maternal DNAm level of each DMR and maternal biochemical parameters, fetal biometric measurements and neonate anthropometry after controlling for confounding factors are shown in Table 2.

**Table 2 -:** Associations between maternal DNA methylation and maternal biochemical parameters, biometric measurements of the fetus, and anthropometry of the neonates.

	Β	r²	p	95% CI
**T3 Abdominal circumference**				
*DMR 3*	-24.266	0.679	**0.006**	-39.724; -8.808
Pre-pregnancy BMI	0.420		0.178	-0.231; 1.071
Gestational weight gain	0.152		0.043	0.006; 0.297
Maternal age	-0.039		0.561	-0.186; 0.107
Neonate sex	-0.016		0.976	-1.198; 1.165
Gestational age	0.658		0.118	-0.202; 1.518
*DMR 5*	-21.559	0.653	**0.009**	-36.275; -6.842
Pre-pregnancy BMI	0.492		0.151	-0.216; 1.201
Gestational weight gain	0.081		0.239	-0.064; 0.225
Maternal age	-0.034		0.626	-0.186; 0.118
Neonate sex	0.896		0.167	-0.451; 2.242
Gestational age	1.026		0.035	0.090; 1.962
*DMR 9*	-25.473	0.518	**0.046**	-50.323; -0.624
Pre-pregnancy BMI	-0.141		0.658	-0.836; 0.555
Gestational weight gain	0.131		0.127	-0.045; 0.308
Maternal age	0.011		0.890	-0.169; 0.192
Neonate sex	0.732		0.321	-0.846; 2.311
Gestational age	0.439		0.377	-0.629; 1.507
***T3* Head circumference**				
*DMR 5*	-4.786	0.961	**0.001**	-6.136; -3.435
Pre-pregnancy BMI	0.090		0.012	0.025; 0.155
Gestational weight gain	0.026		0.002	0.013; 0.039
Maternal age	0.005		0.467	-0.009; 0.019
Neonate sex	0.175		0.011	0.051; 0.298
Gestational age	0.341		0.000	0.255; 0.427
***T4* Head circumference**				
*DMR 1*	7.852	0.730	**0.023**	1.369; 14.335
Pre-pregnancy BMI	0.425		0.081	-0.065; 0.915
Gestational weight gain	0.112		0.050	0.000; 0.225
Maternal age	-0.036		0.358	-0.121; 0.048
Neonate sex	0.267		0.521	-0.637; 1.171
Gestational age	0.322		0.058	-0.013; 0.656
***T2* Fasting insulin**				
*DMR 2*	-20.210	0.903	**0.001**	-28.229; -12.191
Pre-pregnancy BMI	0.669		0.023	0.118; 1.220
Gestational weight gain	0.395		0.000	0.258; 0.532
Maternal age	0.188		0.017	0.043; 0.334
Neonate sex	-2.305		0.006	-3.750; -0.860
Gestational age	-0.659		0.015	-1.154; -0.165
*DMR 6*	-45.581	0.804	**0.008**	-75.799; -15.363
Pre-pregnancy BMI	0.665		0.092	-0.131; 1.461
Gestational weight gain	0.522		0.001	0.288; 0.756
Maternal age	0.177		0.083	-0.029; 0.383
Neonate sex	-2.888		0.015	-5.058; -0.718
Gestational age	-0.876		0.026	-1.620; -0.132
**T1 Triglycerides**				
*DMR2*	-245.651	0.608	**0.034**	-468.802; -22.501
Pre-pregnancy BMI	6.646		0.353	-8.701; 21.993
Gestational weight gain	3.540		0.087	-0.635; 7.715
Maternal age	1.229		0.401	-1.926; 4.384
Neonate sex	-22.645		0.153	-55.468; 10.178
Gestational age	3.890		0.523	-9.357; 17.136
***T2* Triglycerides**				
*DMR 2*	-250.430	0.761	**0.029**	-469.322; -31.538
Pre-pregnancy BMI	19.867		0.015	4.829; 34.906
Gestational weight gain	4.418		0.025	0.680; 8.157
Maternal age	1.808		0.330	-2.164; 5.781
Neonate sex	-30.450		0.115	-69.882; 8.983
Gestational age	1.135		0.853	-12.372; 14.643
***T3* Triglycerides**				
*DMR 9*	-1468.914	0.804	**0.001**	-2126.408; -1.419
Pre-pregnancy BMI	12.552		0.157	-5.846; 30.951
Gestational weight gain	4.693		0.049	0.017; 9.369
Maternal age	2.469		0.271	-2.299; 7.237
Neonate sex	3.075		0.871	-38.696; 44.846
Gestational age	-9.943		0.447	-38.205; 18.319

Multiple linear regression models. DMR: differentially methylated regions. BMI: Body Mass Index. T1 = ≤ 15 gestational weeks. T2 = 20-26 weeks. T3 = 30-36 weeks and T4 = delivery. Confounding factors: pre-pregnancy BMI, gestational weight gain, maternal age, neonate sex and gestational age.

### Integrative network analysis of differentially methylated regions

Functional annotation of the DMRs revealed important biological processes. We highlight the following gene ontology (GO) terms: regulation of DNA methylation, regulation of gene expression by genetic imprinting, regulation of mammary gland epithelial cell proliferation, embryo development, regulation of extrinsic apoptotic signaling pathway, regulation of vascular endothelial growth factor production, regulation of angiogenesis, regulation of vasculature development and regulation of fatty acid biosynthetic process, fatty acid metabolic process, intracellular lipid transport, long-chain fatty acid transport, and fatty acid beta-oxidation The 142 biological processes identified by functional annotation of the DMRs can be found in Table S1.

Regarding molecular functions, we highlight the following GO terms: miRNA binding, integrin binding involved in cell-matrix adhesion, nuclear export signal receptor activity, peptide antigen binding, extracellular matrix constituent conferring elasticity, and carnitine O-palmitoyltransferase activity. The 21 molecular functions resulting from functional annotation of the DMRs can be found in Table S2.

Finally, regarding cellular components, we highlight the following GO terms: protein complex involved in cell adhesion, integrin complex, *EMILIN* complex, MHC protein complex, *BRCA1-A* complex, and ER to Golgi transport vesicle membrane. The 17 cellular components identified by functional annotation of the DMRs can be found in Table S3.

**Figure 3 -:**
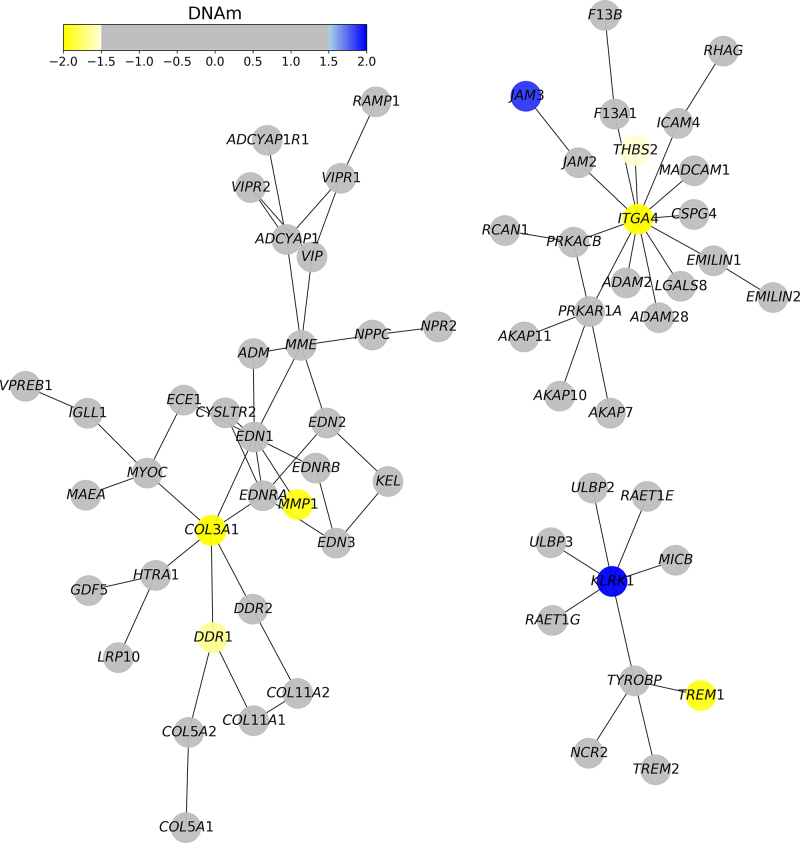
Differentially methylated gene modules around genes *COL3A1*, *ITGA4* and *KLRK1*. Genes are colored by the differential methylation statistics. Differentially methylated gene modules were identified by FEM package (Jiao *et al*., 2014).

In addition, a protein-protein interaction network based on CpG sites located in the promoter region of the genes identified a DMGM in collagen type III alpha 1 chain (*COL3A1*) containing 33 genes, a module in integrin alpha 4 (*ITGA4*) with 21 genes and another module in killer cell lectin like receptor K1 (*KLRK1*) containing 10 genes. The three DMGMs can be seen in Figure 3. Enrichment analysis of DMGMs showed that the *COL3A1* module was related to 21 biological processes and 8 diseases, focused on regulation of blood pressure and cardiovascular and hypertensive diseases. The *ITGA4* module related to 21 biological processes and 5 diseases, targeting to blood coagulation diseases and cell adhesion. The *KLRK1* module was related to 36 biological processes and 3 diseases, targeting susceptibility to natural killer cell-mediated cytotoxicity, leukocyte mediated immunity and innate immune response and diseases involving infection. The biological processes and diseases related to the gene sets are shown in Figure S2 and Figure S3, respectively**.** The functional annotation of each DMGM showed that the three modules are involved in biological processes such as metabolic process and developmental process. *COL3A1* and *ITGA4* modules are also involved in biological processes of reproduction and growth. We highlight that carbohydrate binding appeared as a molecular function in *ITGA4* and lipid binding in *KLRA1*. The complete functional annotation of each DMGM can be found in Figure S4.

## Discussion 

In this study, we observed DMRs and DMGMs enriched for biological processes, cellular components, and molecular functions resulting from EGWG. There was an influence of EGWG on maternal biochemical parameters and fetal and neonate growth parameters, and these parameters were correlated with the mean maternal DNAm level. We found statistically significant differences in TC and TG between the two groups but not in the other parameters, probably because the pregnant women in the two groups investigated were healthy and had a similar and adequate pre-pregnancy BMI. The literature has also shown the influence of altered pre-pregnancy BMI on the lipid profile of pregnant women (Gulecoglu Onem et al., 2021). Furthermore, excessive body weight is known to be a risk factor for increased serum cholesterol levels (Veghari et al., 2015). We found changes due to the effect of time for FG, TC and TG, and higher values of TC and TG at T2 in the EGWG group compared to the AGWG group. Gulecoglu Onem *et al*. (2021) also reported an effect of gestational weight gain on maternal TG and TC which were higher in the second trimester compared to the first trimester (Gulecoglu Onem *et al*., 2021).

Although a relationship between EGWG and the birth of large-for-gestational age babies has been reported in the literature, we did not find a difference in weight or length. However, we observed a difference in an important parameter of fetal growth, OFD at T3, which was higher in the EGWG group compared to the AGWG group. In addition, the HC at birth was greater in the EGWG group compared to AGWG. This finding may be explained by a compensatory mechanism at the end of the last gestational trimester. Moreover, epigenetic marks may have occurred in fetal growth parameters but manifested only at birth. Furthermore, in this study, the interval between T3 and T4 was 6 gestational weeks or more, with the literature reporting significant fetal growth at the end of pregnancy (Champion and Harper, 2020). Analysis of maternal BMI and fetal growth in women at 28 and 36 weeks of gestation showed that a BMI > 40 kg/m^2^ altered fetal growth, with the observation of a lower HC compared to pregnant women with adequate maternal BMI at the two gestational time points investigated (O’Brien et al., 2020). At birth (T4), we found higher TP in the EGWG group compared to AGWG. The PREOBE cohort study also reported a higher TP at birth in obese pregnant women (Berglund et al., 2016). We therefore suggest that EGWG may be as harmful as maternal obesity to neonate anthropometric parameters.

Maternal DNAm was associated with maternal biochemical parameters, including FI at T2 and TG at T1, T2 and T3, in two different DMRs. Microarrays of DNAm have identified epigenetically regulated lipid-related genes in obese patients with hypercholesterolemia (Płatek et al., 2020). Study indicates a supposed causal association between TG and altered DNAm (Ma *et al*., 2020), but there are still no data in the literature that could be compared with our results. Although DNAm was used in this study more as a biomarker of gestational weight gain rather than as an effective factor in fetal development, DNAm in maternal blood may be linked to offspring development according to the Developmental Origins of Health and Disease (DOHaD) theory. This theory explains how the environment early in life can increase the risk of chronic diseases from childhood to adulthood (Barker, 1998). Epigenetic modifications, such as DNAm, are involved in the mediation of how the environment early in life affects later health (Samblas et al., 2017). Thus, gestational weight gain as an environmental factor can alter maternal DNAm and be involved in the mediation of how this environmental factor early in life would affect the phenotype of the offspring, in this case growth.

In the present study, we found an association of DNAm with fetal and neonate growth parameters. The AC at T3 was associated with three different DMRs and HC at T3 and T4 with one DMR. In another study from our research group, maternal DNAm was associated with fetal subcutaneous thigh and arm fat and with neonatal fat mass (Argentato et al., 2023). The literature had reported an association between DNAm and fetal growth (Koukoura et al., 2012). However, to the best of our knowledge, this is the first cohort study that analyzed the association between mean maternal DNAm level in pregnant women with EGWG and several growth parameters at two time points during pregnancy and at birth.

Although we did not test the neonates’ DNA for the DMRs identified here, studies that evaluated obesity and EGWG using similar methodologies found an association with altered methylation patterns in the offspring (Sharp et al., 2015; Takali *et al*., 2017; Mas-Parés et al., 2023). In the Avon study, gestational weight gain was not associated with DNAm in umbilical cord blood of the offspring; however, relatively few women in that study had EGWG (Sharp *et al*., 2015). On the other hand, Thakali et al. (2017) found that gestational weight gain was significantly associated with umbilical cord methylation of IGFBP1. Mas-Parés *et al*. (2023) validated the main methylated CpG loci using pyrosequencing and demonstrated an association of *SETD8*, *SLIT3* and *RPTOR* methylation with gestational weight gain, with higher levels of *SETD8* and *RPTOR* methylation being associated with a higher risk of obesity in the offspring.

Functional annotation provides important information for the biological interpretation of cytosine methylations in genes. There is great interest in determining whether the set of DMRs is enriched for biological processes, molecular functions, and cellular components. Next, we discuss the GOs that may have influenced the weight gain of pregnant women and offspring growth in this study since the literature shows a relationship between the GOs cited below and metabolism. An important GO identified in the present study was the *EMILIN* complex. *EMILINs* are extracellular matrix glycoproteins with regulatory functions in cell migration, differentiation, and proliferation (Zacchigna et al., 2006). The nuclear ubiquitin ligase complex is involved in the muscle atrophy program through the control of signal transduction and modulation of energy balance (Bodine and Baehr, 2014). Downregulation of extracellular matrix constituent, which confers elasticity, has been shown to be associated with worsening of insulin resistance in adipose tissue in obesity (Ruiz-Ojeda et al., 2019).

O-acyl transferase in the liver catalyzes the formation of cholesteryl esters from cholesterol and the activity of carnitine O-palmitoyl transferase, a mitochondrial enzyme, is negatively associated with the muscle content of lipid intermediates (Seiler et al., 2014). Plasma membrane protein complex may also be related to lipid metabolism since fatty acid influx is mediated by an apical heterotetrameric plasma membrane protein complex of which calcium-independent membrane phospholipase A2 (*iPLA2ß*) is a component. Blocking this phospholipase can structurally disrupt the fatty acid uptake complex (Stremmel et al., 2017). Endoplasmic reticulum (ER) to Golgi transport vesicle membrane is involved in lipid biosynthesis, calcium storage, and protein processing. The chronic hyperglycemia and hyperlipidemia associated with type 2 diabetes disrupt ER homeostasis (Mustapha et al., 2021).

MiRNA binding has been implicated in metabolic diseases (Landrier et al., 2019) and even in the gut microbiota in human obesity (Assmann et al., 2020). BRCA1-A complex, a tumor suppressor, has been associated with ovarian (Alkailani et al., 2021) and breast (Bai et al., 2021) cancer in women, and adipocytes can alter the expression of this gene (Tianyi et al., 2018). Synaptonemal complex, a protein structure which is formed between homologous chromosomes during meiosis, can be altered in obesity since a high-fat diet, in addition to inducing obesity in mice, altered the quality of meiosis in oocytes (Yun et al., 2019).

Component integrin alpha4-beta1 complex, which is expressed in cells of the immune system, is involved in cell adhesion, helping to recruit leukocytes to tissue that requires inflammation (Saini et al., 1997). Another GO containing integrins was integrin binding involved in cell-matrix adhesion, which has been shown to be involved in extracellular matrix remodeling, interacting with insulin receptors and modulating insulin sensitivity in white adipose tissue and brown fat thermogenesis (Ruiz-Ojeda et al., 2021). Integrin complex was also involved in adipogenesis in a study on critical signaling factors. In the absence of the integrin complex, insulin growth factor 1 (IGF-1) signals through substrate 1 of the insulin response, inducing sustained AKT signaling and phosphorylation and nuclear exportation of glycogen synthase kinase 3 beta (GSK3b) (Choi et al., 2020).

We found many inflammation-related GOs such as MHC class I protein complex, peptide antigen binding, antigen binding, and protein complex involved in cell adhesion. It is known that chronic inflammation of visceral adipose tissue occurs in obesity (Nishimura et al., 2009) and dysregulation of adipose tissue-resident immune cells in obesity and type 2 diabetes mellitus has been well documented (Lu et al., 2019). A recent study showed that obesity reshapes visceral fat-derived MHC I associated-immunopeptidomes and generates antigenic peptides that drive CD8+ T cell (Chen et al., 2020). Transcription regulatory region DNA binding, like the peroxisome proliferator (PPAR)γ, a transcription factor described in obesity, is involved in adipose tissue differentiation, lipogenesis, and lipid metabolism (Huang et al., 2018).

Three DMGMs found in the analysis of the protein-protein interaction (PPI) network with EGWG shared biological processes involved in metabolism. One module exhibited lipid binding as a molecular function and the other carbohydrate binding. Enrichment analysis showed that these modules are related to diseases that involve cell adhesion, to the innate immune response, and to cardiovascular and hypertensive diseases, providing biological insights into weight gain during pregnancy. PPI networks have been studied in chronic diseases such as obesity (Chen et al., 2017; Chen *et al*., 2021) and in gestational diabetes mellitus (Zhu et al., 2020) but have not yet been described in maternal EGWG.

In a previous study (Argentato et al., 2023), we found subtle changes in the DNAm status of mothers with EGWG in 11 DMRs located in EMILIN1, HOXA5, CPT1B, CLDN9, ZFP57, BRCA1, POU5F1, ANKRD33, HLA-B, RANBP17, ZMYND11, DIP2C, and TMEM232. In contrast, other studies evaluating obese individuals reported denser changes (Ling and Rönn, 2019). Although no difference in the epigenetic signature between EGWG versus obesity prior to pregnancy has been described in the literature, such difference in the epigenetic results could be predicted. However, the methylation results obtained in this study agree with published data on the methylome in obesity, for example an increased risk of type 2 diabetes mellitus due to the effect of methylation on adiposity. Within this context, in a study using computational analyses of obesity (enrichment and pathway analyses), functional categories of GO terms such as “glucose homeostasis” and “glucose response” indicated that genes associated with obesity may confer risks for type 2 diabetes mellitus (Cheng et al., 2018). Another study that sought to identify the methylation profile in DNAm datasets obtained from the GEO database in obesity found methylation-regulated differentially expressed genes, which are involved in the molecular function of fatty acids (Duarte et al., 2023), as highlighted in this study.

This study has some limitations, such as the small sample size and DNAm analysis assessed at the end of pregnancy and not compared to DNAm at baseline. Furthermore, we did not perform DNAm validation. However, our results contribute to an epigenetic understanding of the involvement of an excessive maternal weight gain in the absence of a previous high pre-pregnancy BMI in maternal metabolism, fetal growth, and neonate anthropometry. 

## Conclusion

In conclusion, this study has indicated that DNAm was associated with maternal TG and FI and fetal AC at the end of pregnancy and with HC of the offspring at the end of pregnancy and at birth. The analysis of functional annotation and enrichment of DMGMs revealed processes such as cell division, embryonic development, genomic imprinting, epigenetic mechanisms, inflammation, and lipid and carbohydrates metabolism, which possibly support the maternal-fetal interface. Within this context, the present integrative network analysis of DMRs provided insight how EGWG can potentially trigger changes in biochemical parameters during pregnancy, maternal DNAm and offspring growth. Analysis of DNAm may in the future become a tool for identifying risks for maternal metabolism and maternal diseases in pregnancy such as hypertension.

## Supplementary material

The following online material is available for this article:

Table S1 - Biological processes.

Table S2 - Molecular functions.

Table S3 - Cellular components.

Figure S1 - Evolution of the offspring growth.

Figure S2 - Enriched biological processes (p-value<0.05) for the differentially methylated gene modules.

Figure S3 - Enriched diseases (p-value<0.05) for the differentially methylated gene modules.

Figure S4 - Functional annotation of the differentially methylated gene modules.
