# Supplementary material for: Integrative network analysis of differentially methylated regions to study the impact of gestational weight gain on maternal metabolism and fetal-neonatal growth
Source: Genet Mol Biol. 2024 Mar 25;47(1):e20230203. doi: 10.1590/1678-4685-GMB-2023-0203 (PMC10993311; doi:10.1590/1678-4685-GMB-2023-0203)
Supplement: Table S1 - [file 1415-4757-GMB-47-1-e20230203-s1.pdf]

Genetics and Molecular Biology
https://doi.org/10.1590/1678-4685-GMB-2023-0203

Supplementary Material to "Integrative network analysis of differentially methylated regions to study the impact of gestational weight gain on maternal metabolism and fetal-neonatal growth"

Table S1 - Gene ontology (GO) of biological processes of women with excess gestational weight gain versus adequate gestational weight gain.

GO Biological Processes	Binom Raw P-Value	Binom Fold Enrichment
Positive regulation of histone H4-K20 methylation	5.3E-04	1,883.92
Cell-cell signaling involved in mammary gland development	7.4E-04	1,357.10
Lung goblet cell differentiation	1.2E-03	814.71
Lobar bronchus epithelium development	1.4E-03	689.40
Intestinal epithelial cell maturation	2.4E-03	417.38
Lobar bronchus development	3.3E-03	305.98
Regulation of asymmetric cell division	3.8E-03	263.73
Antigen processing and presentation of exogenous peptide antigen via MHC class I, TAP-independent	3.8E-03	260.50
DNA methylation involved in embryo development	4.9E-03	202.33
Trachea cartilage morphogenesis	5.8E-03	172.49
Regulation of gene expression by genetic imprinting	8.7E-05	144.12
Bronchiole development	7.4E-03	135.56
Positive regulation of receptor biosynthetic process	7.6E-03	130.49
Negative regulation of histone H3-K4 methylation	7.8E-03	128.54
Negative regulation of centriole replication	7.8E-03	127.14
Positive regulation of histone H3-K9 acetylation	8.0E-03	123.86
Cellular response to indole-3-methanol	8.2E-03	122.21
Positive regulation of histone H4-K16 acetylation	8.2E-03	121.66
Protein K6-linked ubiquitination	8.9E-03	111.55
Positive regulation of histone H4 acetylation	9.7E-03	103.15
Negative regulation of centrosome duplication	9.7E-03	102.27
mRNA transcription from RNA polymerase II promoter	9.9E-03	100.22
Genetic imprinting	1.8E-04	99.75
Negative regulation of fatty acid biosynthetic process	1.0E-02	98.85
Negative regulation of histone H3-K9 methylation	1.0E-02	95.84
Negative regulation of centrosome cycle	1.1E-02	93.11
Regulation of histone H4-K16 acetylation	1.1E-02	92.93
Carnitine shuttle	1.1E-02	92.51
Regulation of histone H3-K9 acetylation	1.1E-02	91.88
Fatty acid transmembrane transport	1.1E-02	91.14
Positive regulation of histone H3-K9 methylation	1.1E-02	90.88
Respiratory system process	1.2E-02	84.27
DNA damage response, signal transduction by p53 class mediator resulting in transcription of p21 class mediator	1.2E-02	81.07

GO Biological Processes	Binom Raw P-Value	Binom Fold Enrichment
columnar/cuboidal epithelial cell maturation	1.2E-02	80.54
DNA damage response, signal transduction resulting in transcription	1.2E-02	80.28
mRNA transcription	1.3E-02	78.63
Negative regulation of erythrocyte differentiation	1.3E-02	77.67
Dosage compensation by inactivation of X chromosome	1.3E-02	77.30
Regulation of histone H4 acetylation	1.3E-02	75.67
Endodermal cell fate specification	1.4E-02	73.56
negative regulation of histone acetylation	1.4E-02	73.28
Regulation of receptor biosynthetic process	1.4E-02	70.93
Trachea cartilage development	1.4E-02	70.56
Calcium-independent cell-cell adhesion via plasma membrane cell-adhesion molecules	1.5E-02	64.76
Intestinal epithelial cell development	1.6E-02	63.83
Dosage compensation	1.6E-02	63.43
Negative regulation of gene silencing by miRNA	1.6E-02	63.26
Regulation of centriole replication	1.6E-02	63.23
Negative regulation of fatty acid metabolic process	1.6E-02	61.77
Regulation of transcription elongation from RNA polymerase II promoter	1.6E-02	61.73
Negative regulation of peptidyl-lysine acetylation	1.6E-02	61.24
Lung secretory cell differentiation	1.7E-02	59.81
Negative regulation of protein acetylation	1.7E-02	59.26
Protein export from nucleus	1.7E-02	58.09
Negative regulation of posttranscriptional gene silencing	1.7E-02	57.42
Negative regulation of gene silencing by RNA	1.7E-02	57.42
Regulation of DNA methylation	1.8E-02	54.01
Protein homotrimerization	2.0E-02	50.38
Mitotic G2/M transition checkpoint	2.0E-02	49.02
Bronchus development	2.1E-02	47.18
Negative regulation of intracellular estrogen receptor signaling pathway	2.1E-02	46.26
Intestinal epithelial cell differentiation	2.2E-02	45.38
Intracellular lipid transport	2.2E-02	44.97
Thyroid gland development	2.2E-02	44.73
Regulation of histone H3-K9 methylation	2.3E-02	43.94
DNA double-strand break processing	2.3E-02	43.71
Positive regulation of histone H3-K4 methylation	2.3E-02	43.29
Mesenchymal-epithelial cell signaling	2.3E-02	43.17
Endodermal cell fate commitment	2.3E-02	42.32
Negative regulation of histone methylation	2.4E-02	40.78
Negative regulation of organelle assembly	2.5E-02	39.83
Epithelial cell maturation	2.6E-02	38.67
Regulation of mammary gland epithelial cell proliferation	2.6E-02	38.10
Cartilage morphogenesis	2.6E-02	37.92
Strand displacement	2.6E-02	37.87
G2 DNA damage checkpoint	2.6E-02	37.53
Type I interferon signaling pathway	2.6E-02	37.33
Lung-associated mesenchyme development	2.7E-02	37.13
Negative regulation of gene silencing	2.7E-02	37.12
Trachea morphogenesis	2.7E-02	37.07

GO Biological Processes	Binom Raw P-Value	Binom Fold Enrichment
Regulation of DNA-templated transcription, elongation	2.7E-02	36.50
Response to type I interferon	2.9E-02	34.11
Negative regulation of transcription regulatory region DNA binding	2.9E-02	33.81
Regulation of transcription from RNA polymerase III promoter	2.9E-02	33.53
Protein trimerization	3.0E-02	33.21
Negative regulation of extrinsic apoptotic signaling pathway via death domain receptors	3.0E-02	33.09
Mammary gland epithelial cell differentiation	3.3E-02	29.61
Negative regulation of G0 to G1 transition	3.5E-02	27.77
Regulation of fatty acid biosynthetic process	3.6E-02	27.34
Antigen processing and presentation of exogenous peptide antigen via MHC class I, TAP-dependent	3.6E-02	26.98
Positive regulation of histone methylation	3.7E-02	26.54
Fatty acid beta-oxidation	3.7E-02	26.46
Regulation of histone H3-K4 methylation	3.7E-02	26.27
Antigen processing and presentation of exogenous peptide antigen via MHC class I	3.8E-02	26.13
Protein autoubiquitination	3.8E-02	25.93
Regulation of G0 to G1 transition	3.8E-02	25.88
Negative regulation of JNK cascade	3.8E-02	25.59
Positive regulation of vascular endothelial growth factor production	3.9E-02	25.43
Negative regulation of reactive oxygen species metabolic process	4.0E-02	24.64
Negative regulation of histone modification	4.0E-02	24.32
Postreplication repair	4.1E-02	24.11
Mammary gland alveolus development	4.1E-02	23.84
positive regulation of histone acetylation	4.3E-02	23.03
Regulation of centrosome duplication	4.3E-02	22.78
Trachea development	4.3E-02	22.62
Long-chain fatty acid transport	4.4E-02	22.15
Regulation of vascular endothelial growth factor production	4.4E-02	22.05
DNA methylation	4.6E-02	21.50
Regulation of erythrocyte differentiation	4.6E-02	21.38
Negative regulation of intracellular steroid hormone receptor signaling pathway	4.6E-02	21.25
Double-strand break repair via nonhomologous end joining	4.7E-02	20.62
Intrinsic apoptotic signaling pathway in response to DNA damage	4.8E-02	20.30
Cell fate commitment involved in formation of primary germ layer	4.8E-02	20.23
Positive regulation of peptidyl-lysine acetylation	4.9E-02	20.01
Interferon-gamma-mediated signaling pathway	4.9E-02	19.97
Lung epithelial cell differentiation	4.9E-02	19.97
Negative regulation of chromatin organization	4.9E-02	19.75
Negative regulation of extrinsic apoptotic signaling pathway	7.0E-03	15.55
Regulation of gene expression, epigenetic	1.2E-02	11.59
Regulation of extrinsic apoptotic signaling pathway	1.4E-02	10.91
Negative regulation of apoptotic signaling pathway	2.0E-02	9.07
Fatty acid metabolic process	2.7E-02	7.67
Regulation of angiogenesis	3.5E-02	6.60

GO Biological Processes	Binom Raw P-Value	Binom Fold Enrichment
Viral process	1.3E-02	5.85
Multi-organism cellular process	1.3E-02	5.74
Regulation of vasculature development	4.6E-02	5.72
Symbiosis, encompassing mutualism through parasitism	1.7E-02	5.26
Interspecies interaction between organisms	1.7E-02	5.25
Chordate embryonic development	3.8E-02	3.85
Embryo development ending in birth or egg hatching	3.9E-02	3.83
Negative regulation of nucleic acid-templated transcription	8.8E-03	3.49
Negative regulation of RNA biosynthetic process	9.1E-03	3.46
Negative regulation of RNA metabolic process	1.1E-02	3.33
Embryo development	3.3E-02	3.06
Negative regulation of macromolecule biosynthetic process	1.6E-02	3.03
Negative regulation of nucleobase-containing compound metabolic process	1.7E-02	2.99
Negative regulation of cellular biosynthetic process	2.0E-02	2.87
Negative regulation of biosynthetic process	2.1E-02	2.85
Negative regulation of transcription, DNA-templated	4.2E-02	2.84
Negative regulation of nitrogen compound metabolic process	2.2E-02	2.80
Regulation of transcription from RNA polymerase II promoter	1.6E-02	2.55

Functional annotation of DMRs was performed using GREAT.
