# Supplementary material for: Integrative network analysis of differentially methylated regions to study the impact of gestational weight gain on maternal metabolism and fetal-neonatal growth
Source: Genet Mol Biol. 2024 Mar 25;47(1):e20230203. doi: 10.1590/1678-4685-GMB-2023-0203 (PMC10993311; doi:10.1590/1678-4685-GMB-2023-0203)
Supplement: Table S2 - [file 1415-4757-GMB-47-1-e20230203-s2.pdf]

Genetics and Molecular Biology
https://doi.org/10.1590/1678-4685-GMB-2023-0203

Supplementary Material to "Integrative network analysis of differentially methylated regions to study the impact of gestational weight gain on maternal metabolism and fetal-neonatal growth"

Table S2 - Gene ontology (GO) of molecular functions of women with excess gestational weight gain versus adequate gestational weight gain.

GO Molecular functions	Binom Raw P-Value	Binom Fold Enrichment
Integrin binding involved in cell-matrix adhesion	1.2E-04	8530.01
Carnitine O-palmitoyltransferase activity	1.2E-03	849.80
O-palmitoyltransferase activity	1.8E-03	559.81
Carnitine O-acyltransferase activity	2.3E-03	441.67
Nuclear export signal receptor activity	7.4E-03	134.80
Peptide antigen binding	7.9E-03	126.62
Extracellular matrix constituent conferring elasticity	8.6E-03	116.33
Protein binding involved in cell-matrix adhesion	1.0E-02	96.93
Structural molecule activity conferring elasticity	1.9E-02	51.34
miRNA binding	2.3E-02	42.49
Ran GTPase binding	3.1E-02	32.18
Nucleocytoplasmic transporter activity	3.4E-02	29.33
Palmitoyltransferase activity	3.5E-02	28.49
Antigen binding	4.0E-02	24.80
O-acyltransferase activity	4.2E-02	23.27
RNA polymerase binding	4.6E-02	21.06
Ubiquitin protein ligase binding	3.8E-02	6.38
Ubiquitin-like protein ligase binding	3.9E-02	6.27
Transcription regulatory region DNA binding	2.1E-02	3.50
Regulatory region DNA binding	2.1E-02	3.50
Regulatory region nucleic acid binding	2.1E-02	3.49

Functional annotation of DMRs was performed using GREAT.
