# Supplementary material for: Integrative network analysis of differentially methylated regions to study the impact of gestational weight gain on maternal metabolism and fetal-neonatal growth
Source: Genet Mol Biol. 2024 Mar 25;47(1):e20230203. doi: 10.1590/1678-4685-GMB-2023-0203 (PMC10993311; doi:10.1590/1678-4685-GMB-2023-0203)
Supplement: Table S3 - [file 1415-4757-GMB-47-1-e20230203-s3.pdf]

Genetics and Molecular Biology
https://doi.org/10.1590/1678-4685-GMB-2023-0203

Supplementary Material to "Integrative network analysis of differentially methylated regions to study the impact of gestational weight gain on maternal metabolism and fetal-neonatal growth"

Table S3 - Gene ontology (GO) of cellular component of women with excess gestational weight gain versus adequate gestational weight gain.

GO Cellular component	Binom Raw P-Value	Binom Fold Enrichment
Integrin alpha4-beta1 complex	3.1E-05	31799.84
EMILIN complex	3.1E-05	31799.84
MHC class I protein complex	3.0E-03	329.60
BRCA1-BARD1 complex	3.3E-03	301.26
BRCA1-A complex	5.1E-03	196.29
MHC protein complex	6.3E-03	158.87
Integral component of lumenal side of endoplasmic Reticulum membrane	9.6E-03	103.71
Gamma-tubulin ring complex	1.0E-02	99.32
Lateral element	1.7E-02	59.34
Gamma-tubulin complex	2.0E-02	50.46
Nuclear ubiquitin ligase complex	2.1E-02	47.14
Synaptonemal complex	2.9E-02	34.43
Nuclear heterochromatin	3.0E-02	32.49
Integrin complex	3.2E-02	30.74
Protein complex involved in cell adhesion	3.4E-02	29.14
ER to Golgi transport vesicle membrane	3.9E-02	24.92
Plasma membrane protein complex	2.7E-02	7.67

Functional annotation of DMRs was performed using GREAT.
