# Supplementary material for: Integrative network analysis of differentially methylated regions to study the impact of gestational weight gain on maternal metabolism and fetal-neonatal growth
Source: Genet Mol Biol. 2024 Mar 25;47(1):e20230203. doi: 10.1590/1678-4685-GMB-2023-0203 (PMC10993311; doi:10.1590/1678-4685-GMB-2023-0203)
Supplement: Figure S2 - [file 1415-4757-GMB-47-1-e20230203-s5.pdf]

Genetics and Molecular Biology
https://doi.org/10.1590/1678-4685-GMB-2023-0203

Supplementary Material to "Integrative network analysis of differentially methylated regions to study the impact of gestational weight gain on maternal metabolism and fetal-neonatal growth"

COL3A1

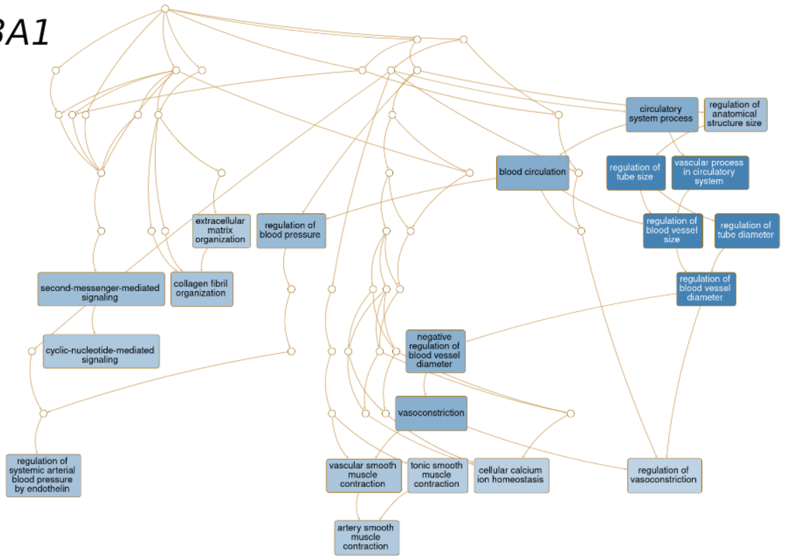

ITGA4

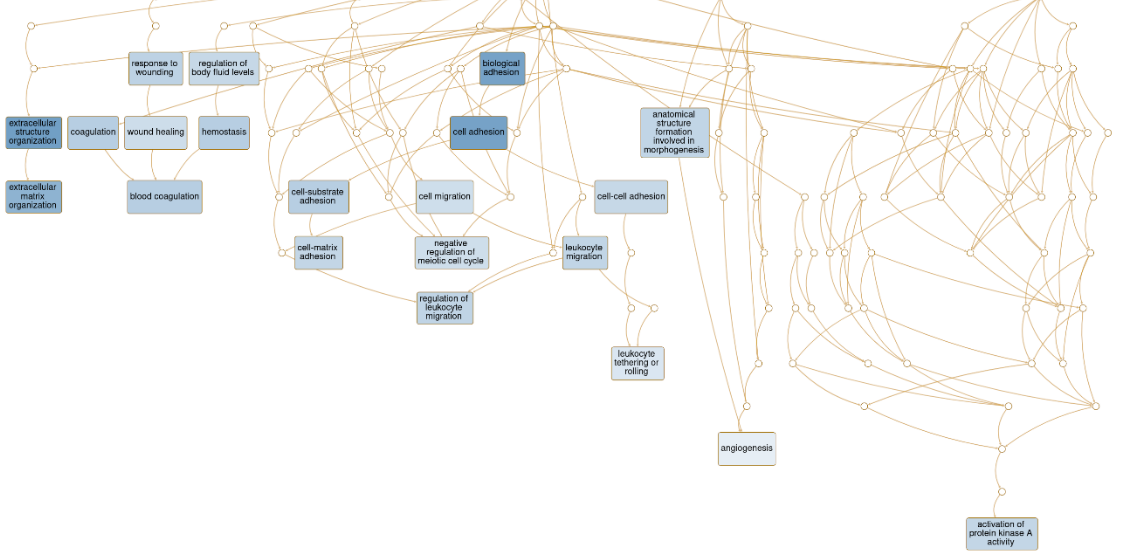

KLKR1

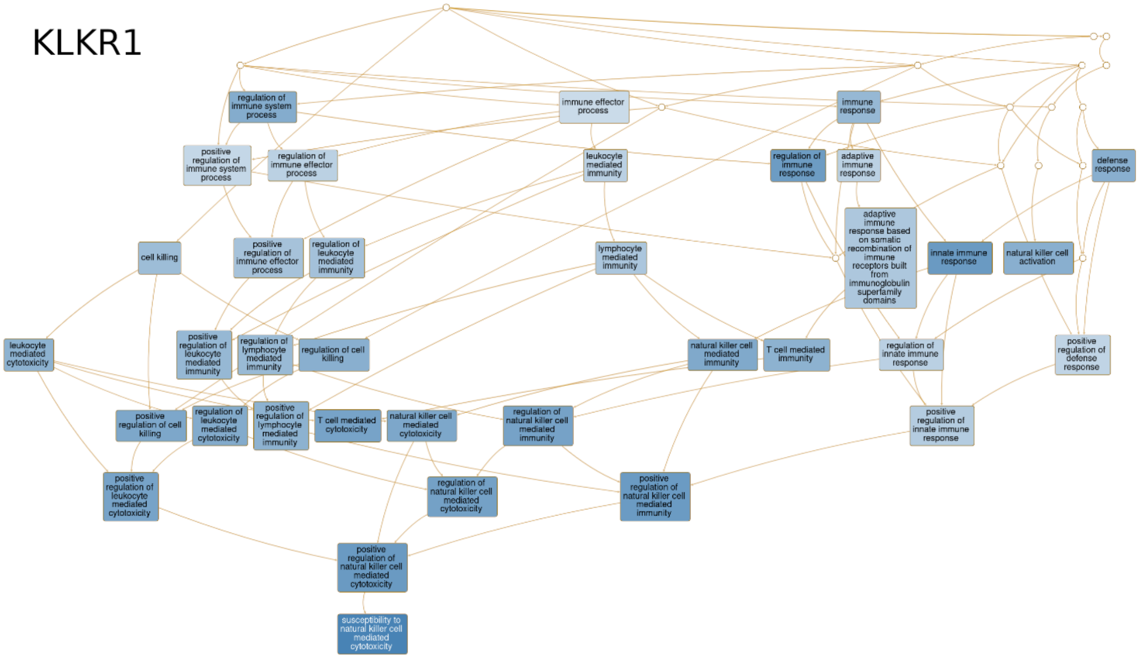

Figure S2 - Enriched biological processes (p-value<0.05) for the differentially methylated gene modules. Enrichment analysis was performed in WebGestalt, considering the Gene Ontology database (Ashburner *et al.*, 2000). The reference set was the genome, corresponding to 61506 Entrez gene IDs with 25166 IDs annotated to the selected functional categories used as the enrichment analysis reference.

Reference

Ashburner M, Ball CA, Blake JA, Botstein D, Butler H, Cherry JM, Davis AP, Dolinski K, Dwight SS, Eppig JT *et al.* (2000) Gene Ontology: Tool for the unification of biology. Nat Genet 25:25-29.
