# Supplementary material for: Integrative network analysis of differentially methylated regions to study the impact of gestational weight gain on maternal metabolism and fetal-neonatal growth
Source: Genet Mol Biol. 2024 Mar 25;47(1):e20230203. doi: 10.1590/1678-4685-GMB-2023-0203 (PMC10993311; doi:10.1590/1678-4685-GMB-2023-0203)
Supplement: Figure S3 - [file 1415-4757-GMB-47-1-e20230203-s6.pdf]

Genetics and Molecular Biology
https://doi.org/10.1590/1678-4685-GMB-2023-0203

Supplementary Material to "Integrative network analysis of differentially methylated regions to study the impact of gestational weight gain on maternal metabolism and fetal-neonatal growth"

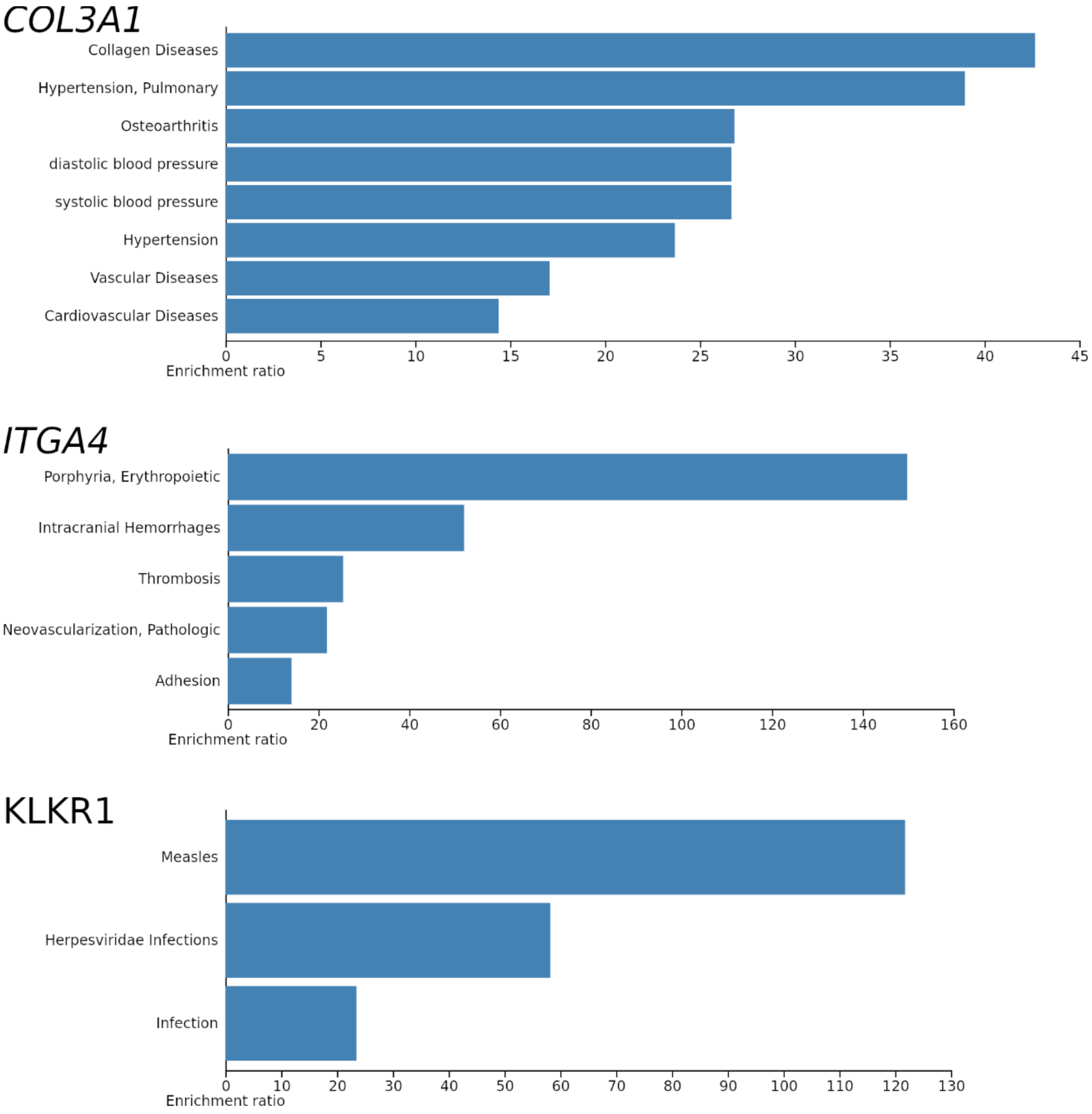

Figure S3 - Enriched diseases (p-value<0.05) for the differentially methylated gene modules. Enrichment analysis was performed in WebGestalt, considering disease terms from the PharmGKB (Whirl-Carrillo *et al.*, 2021) and genes associated with the individual disease were inferred using GLAD4U (Jourquin *et al.*, 2012). The reference set was the genome, corresponding to 61506 Entrez gene IDs with 25166 IDs annotated to the selected functional categories used as the enrichment analysis reference.

References

Jourquin J, Duncan D and Shi Z (2012) GLAD4U: Deriving and prioritizing gene lists from PubMed literature. BMC Genomics 13:S20.

Whirl-Carrillo M, Huddart R, Gong L, Sangkuhl K, Thorn CF, Whaley R, Klein TE (2021) An evidence-based framework for evaluating pharmacogenomics knowledge for personalized medicine. Clin Pharmacol Ther 110:563-572.
