# Supplementary material for: Integrative network analysis of differentially methylated regions to study the impact of gestational weight gain on maternal metabolism and fetal-neonatal growth
Source: Genet Mol Biol. 2024 Mar 25;47(1):e20230203. doi: 10.1590/1678-4685-GMB-2023-0203 (PMC10993311; doi:10.1590/1678-4685-GMB-2023-0203)
Supplement: Figure S4 - [file 1415-4757-GMB-47-1-e20230203-s7.pdf]

Genetics and Molecular Biology
https://doi.org/10.1590/1678-4685-GMB-2023-0203

Supplementary Material to "Integrative network analysis of differentially methylated regions to study the impact of gestational weight gain on maternal metabolism and fetal-neonatal growth"

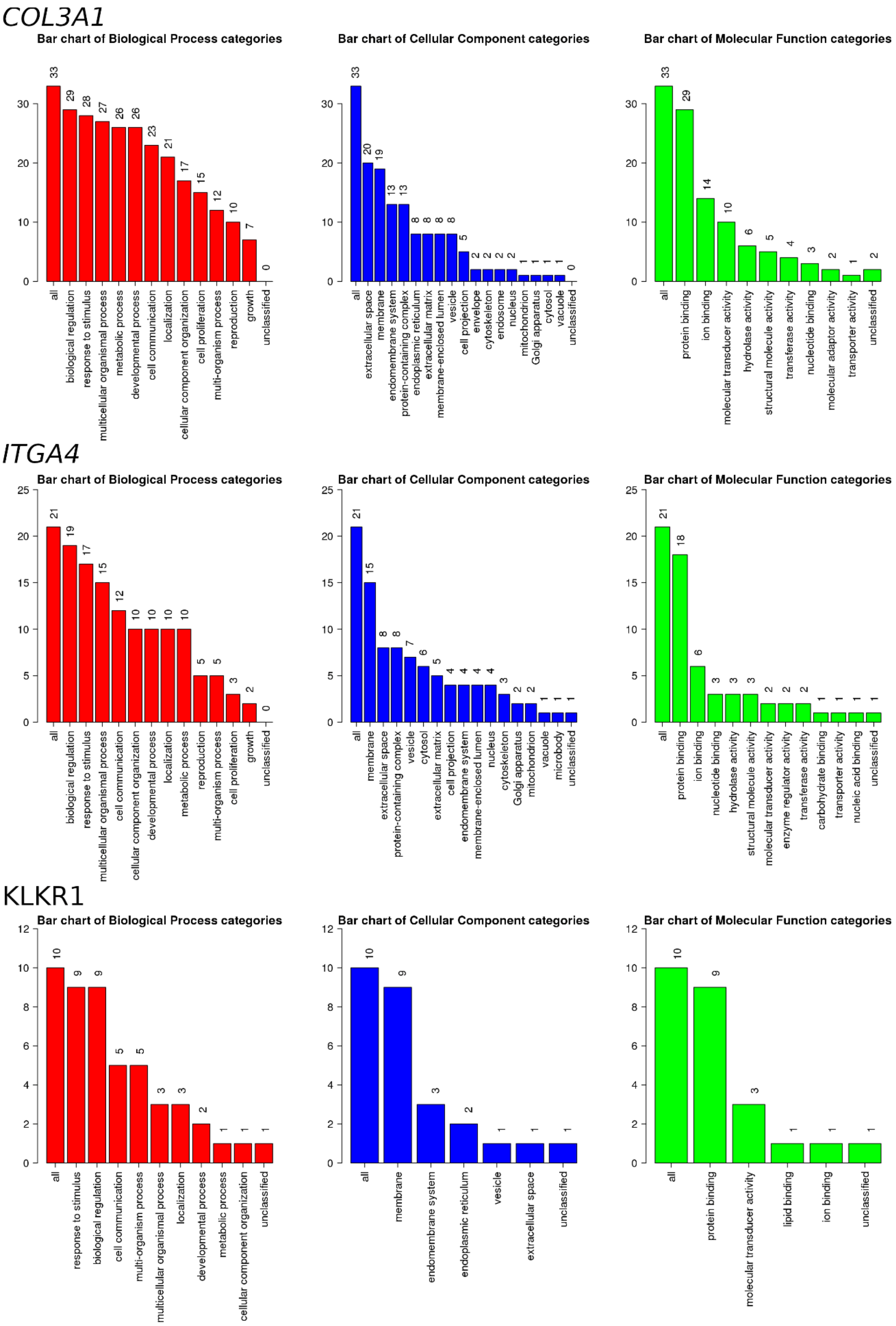

Figure S4 - Functional annotation of the differentially methylated gene modules. Each Biological Process, Cellular Component and Molecular Function category were annotated by WebGestalt (Liao *et al.*, 2019), based on the GO Slim database. The height of the bar chart indicates the number of IDs in the differentially methylated gene modules and also in the category.

Reference

Liao Y, Wang J, Jaehnig EJ, Shi Z and Zhang B (2019) WebGestalt 2019: Gene set analysis toolkit with revamped UIs and APIs. Nucleic Acids Res 47:W199-W205.
